# The genome sequence of the Lobe-spurred Furrow Bee, *Lasioglossum pauxillum *(Schenck, 1853)

**DOI:** 10.12688/wellcomeopenres.20950.1

**Published:** 2024-02-19

**Authors:** Liam M. Crowley, University of Oxford and Wytham Woods Genome Acquisition Lab, Darwin Tree of Life Barcoding collective, Wellcome Sanger Institute Tree of Life Management, Samples and Laboratory team, Wellcome Sanger Institute Scientific Operations: Sequencing Operations, Wellcome Sanger Institute Tree of Life Core Informatics team, Tree of Life Core Informatics collective, Darwin Tree of Life Consortium

**Affiliations:** 1 University of Oxford, Oxford, England, UK

**Keywords:** Lasioglossum pauxillum, Lobe-spurred Furrow Bee, genome sequence, chromosomal, Hymenoptera

## Abstract

We present a genome assembly from an individual female *Lasioglossum pauxillum* (the Lobe-spurred Furrow Bee; Arthropoda; Insecta; Hymenoptera; Halictidae). The genome sequence is 432.0 megabases in span. Most of the assembly is scaffolded into 9 chromosomal pseudomolecules. The mitochondrial genome has also been assembled and is 27.71 kilobases in length. Gene annotation of this assembly on Ensembl identified 12,353 protein coding genes.

## Species taxonomy

Eukaryota; Metazoa; Eumetazoa; Bilateria; Protostomia; Ecdysozoa; Panarthropoda; Arthropoda; Mandibulata; Pancrustacea; Hexapoda; Insecta; Dicondylia; Pterygota; Neoptera; Endopterygota; Hymenoptera; Apocrita; Aculeata; Apoidea; Anthophila; Halictidae; Halictinae; Halictini; *Lasioglossum*; *Evylaeus*; *Lasioglossum pauxillum* (Schenck, 1853) (NCBI:txid88516).

## Background

The Lobe-spurred Furrow Bee, *Lasioglossum pauxillum*, is a small (forewing length 3.5–4.5 mm) dark bee in the family Halictidae. It occurs throughout Europe, from Portugal to Georgia, and into north Africa. In the UK is it common and widespread across southern England and occurs into the Midlands and Wales. The inner spur of the hind tibia of females has broadly rounded projections rather than narrow and pointed teeth, which is unique amongst British *Lasioglossum* species. Females also have a carinate propodeum, pale wing stigmas, a complete ridge defining the apical depression of tergite one. and the antennal flagella is usually orange underneath.

*L. pauxillum* is associated with open habitats, especially chalk grassland where it can be abundant. It is a primitively eusocial species ( Plateaux-Quénu, 2008), with overwintered females emerging from April and producing workers by early summer. Males and gynes are produced from July to October, with mated females overwintering. Nest often have a ‘turret-like’ entrance ( Westrich, 1989) and occur in bare or sparsely vegetated, level soil, in aggregations that can reach large numbers ( Pesenko *et al.*, 2000). Macrocyclic lactones are used as queen recognition signals, regulating worker behaviour and physiology, and therefore maintaining reproductive harmony ( Steitz *et al.*, 2019). A wide range of flowers are visited for nectar, including umbellifers, composites, buttercups, spring-flowering shrubs and especially common fleabane, *Pulicaria dysenterica* ( Falk & Lewington, 2019). It is oligolectic, visiting fewer species of flowers for pollen than other *Lasioglossum* species ( Polidori *et al.*, 2010), but from a taxonomically broad range ( Beil *et al.*, 2008).

The complete genome sequence for this species will facilitate studies into the evolution of sociality, reproductive systems and Hymenopteran taxonomy.

## Genome sequence report

**Figure 1.**
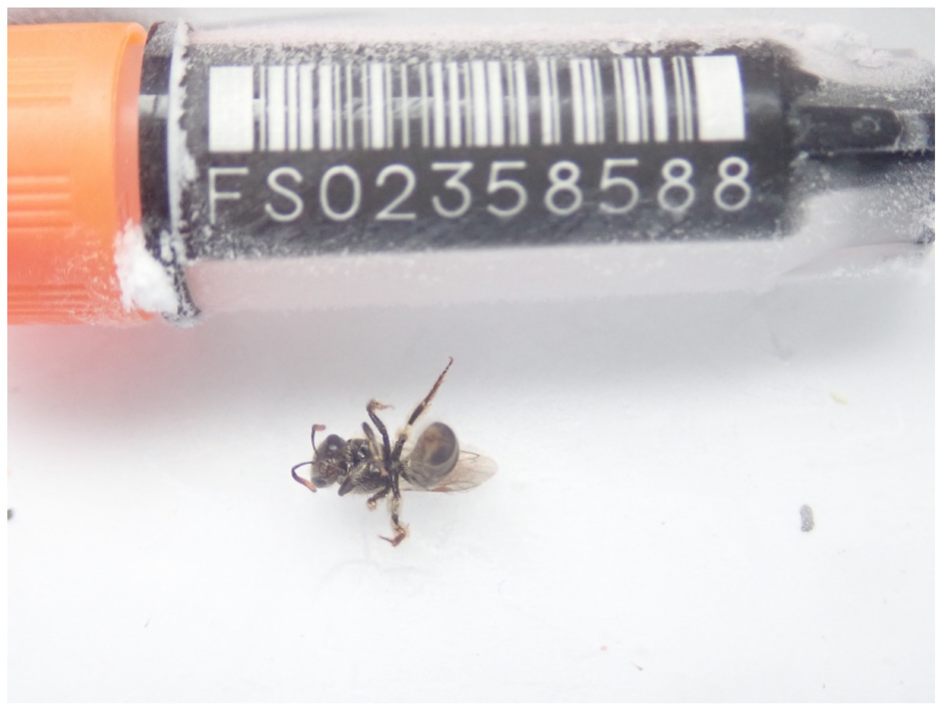
Photograph of the *Lasioglossum pauxillum* (iyLasPaux1) specimen used for genome sequencing.

The genome was sequenced from one female *Lasioglossum pauxillum* ( Figure 1) collected from Wytham Woods, Oxfordshire, UK (51.78, –1.32). A total of 59-fold coverage in Pacific Biosciences single-molecule HiFi long reads was generated. Primary assembly contigs were scaffolded with chromosome conformation Hi-C data. Manual assembly curation corrected 40 missing joins or mis-joins, reducing the scaffold number by 52.05%, and increasing the scaffold N50 by 100.79%.

**Figure 2.**
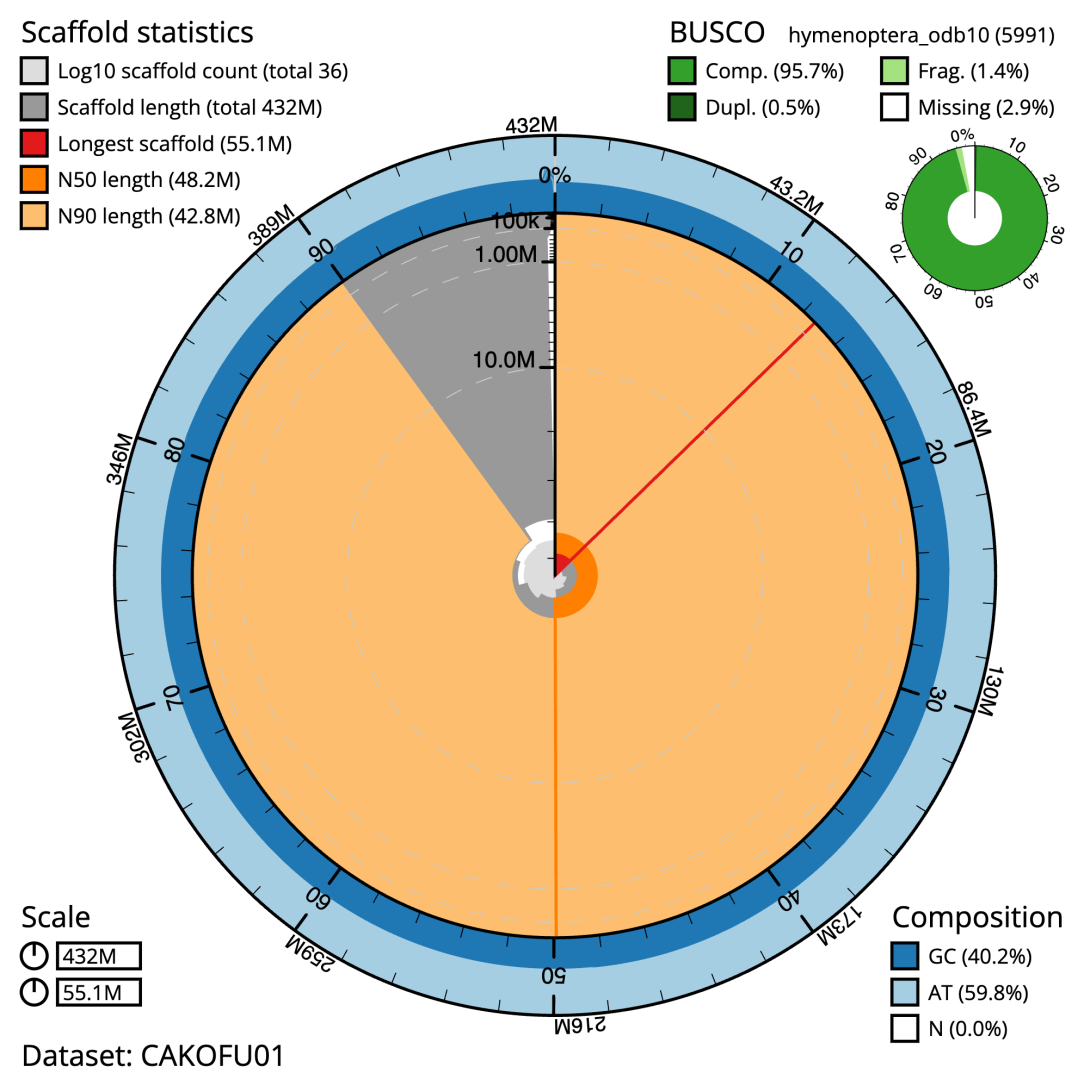
Genome assembly of *Lasioglossum pauxillum*, iyLasPaux1.1: metrics. The BlobToolKit Snailplot shows N50 metrics and BUSCO gene completeness. The main plot is divided into 1,000 size-ordered bins around the circumference with each bin representing 0.1% of the 432,014,419 bp assembly. The distribution of scaffold lengths is shown in dark grey with the plot radius scaled to the longest scaffold present in the assembly (55,061,150 bp, shown in red). Orange and pale-orange arcs show the N50 and N90 scaffold lengths (48,162,910 and 42,843,013 bp), respectively. The pale grey spiral shows the cumulative scaffold count on a log scale with white scale lines showing successive orders of magnitude. The blue and pale-blue area around the outside of the plot shows the distribution of GC, AT and N percentages in the same bins as the inner plot. A summary of complete, fragmented, duplicated and missing BUSCO genes in the hymenoptera_odb10 set is shown in the top right. An interactive version of this figure is available at https://blobtoolkit.genomehubs.org/view/CAKOFU01/dataset/CAKOFU01/snail.

**Figure 3.**
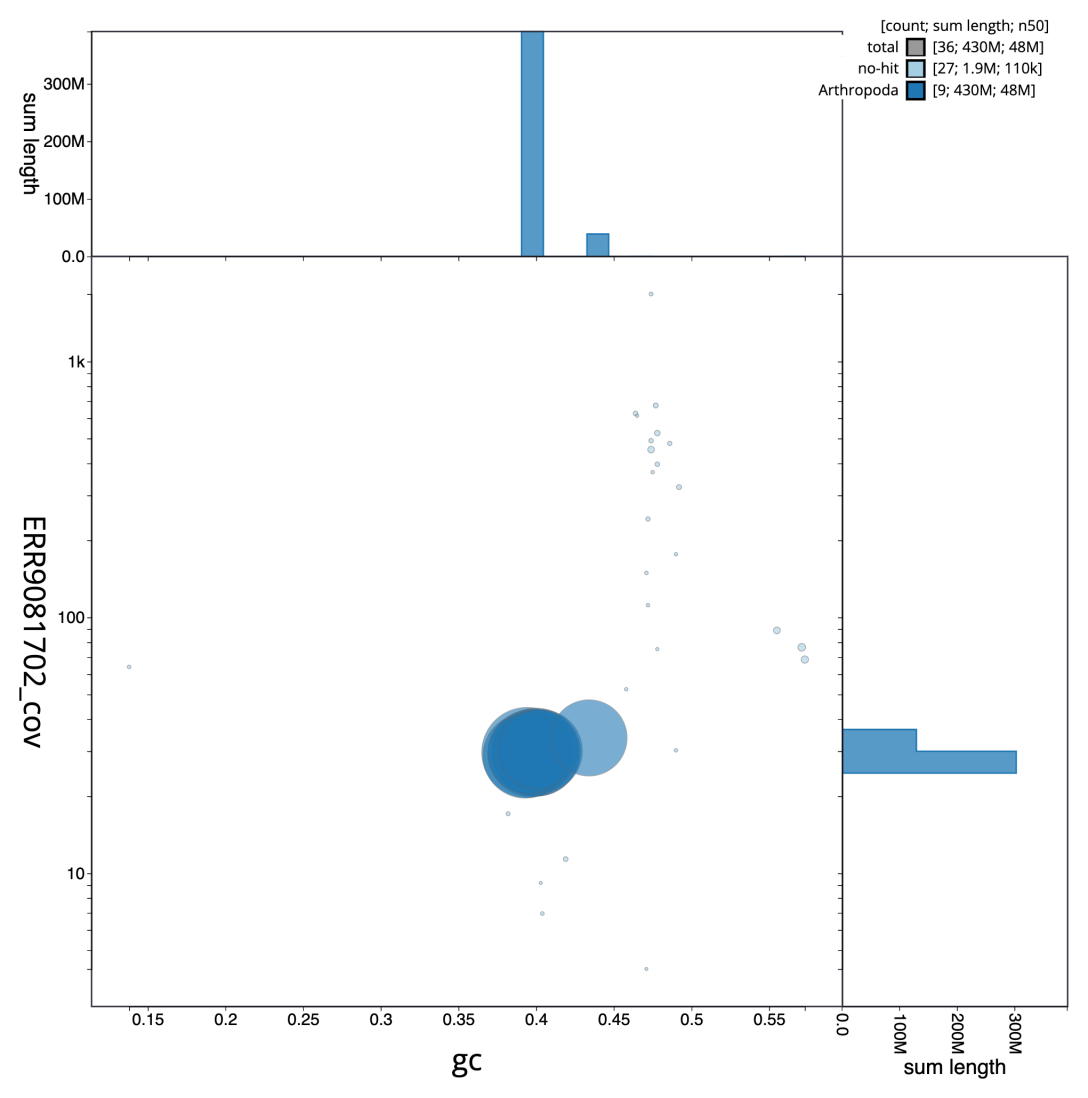
Genome assembly of *Lasioglossum pauxillum*, iyLasPaux1.1: BlobToolKit GC-coverage plot. Scaffolds are coloured by phylum. Circles are sized in proportion to scaffold length. Histograms show the distribution of scaffold length sum along each axis. An interactive version of this figure is available at https://blobtoolkit.genomehubs.org/view/CAKOFU01/dataset/CAKOFU01/blob.

**Figure 4.**
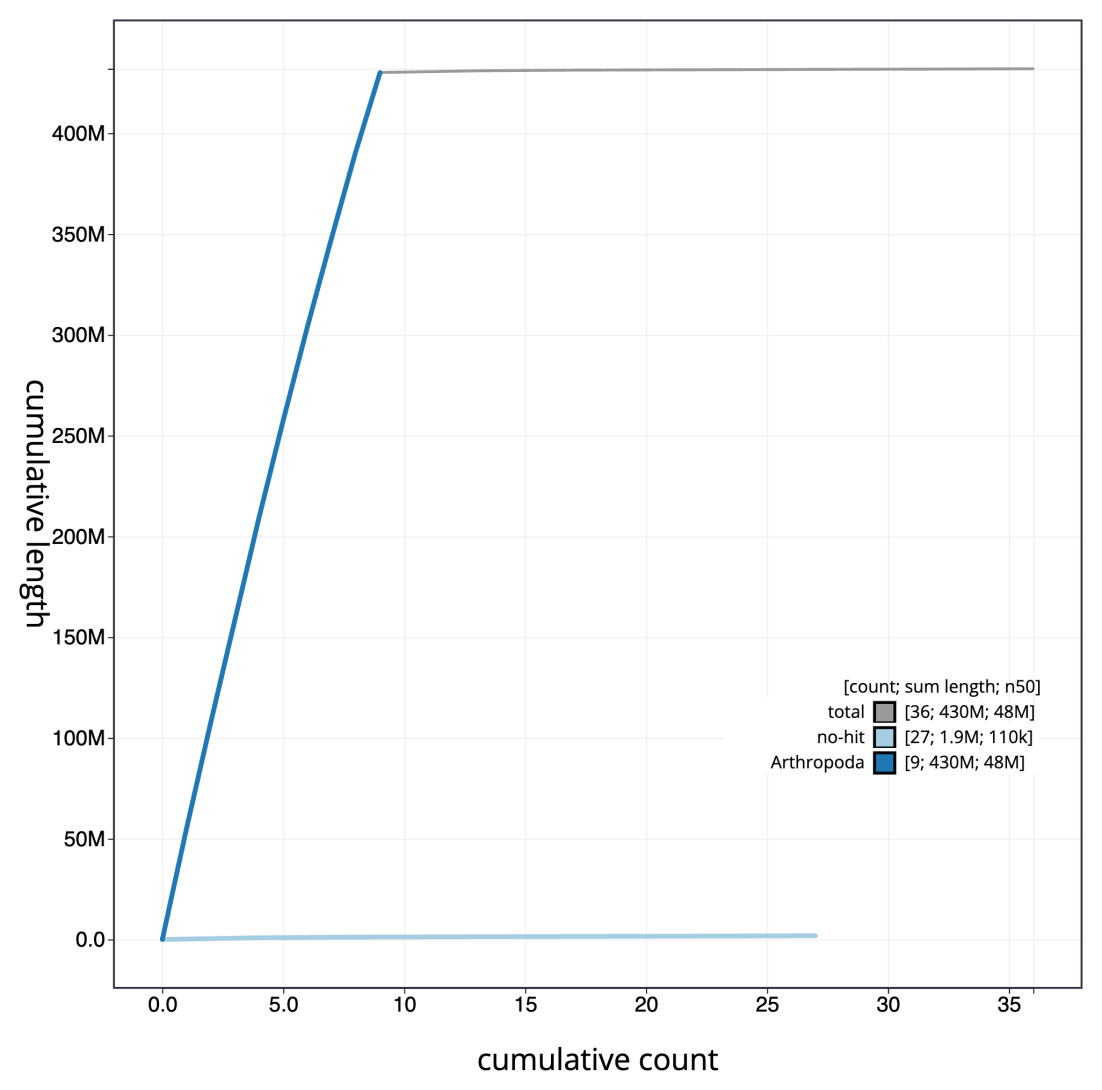
Genome assembly of *Lasioglossum pauxillum*, iyLasPaux1.1: BlobToolKit cumulative sequence plot. The grey line shows cumulative length for all scaffolds. Coloured lines show cumulative lengths of scaffolds assigned to each phylum using the buscogenes taxrule. An interactive version of this figure is available at https://blobtoolkit.genomehubs.org/view/CAKOFU01/dataset/CAKOFU01/cumulative.

**Figure 5.**
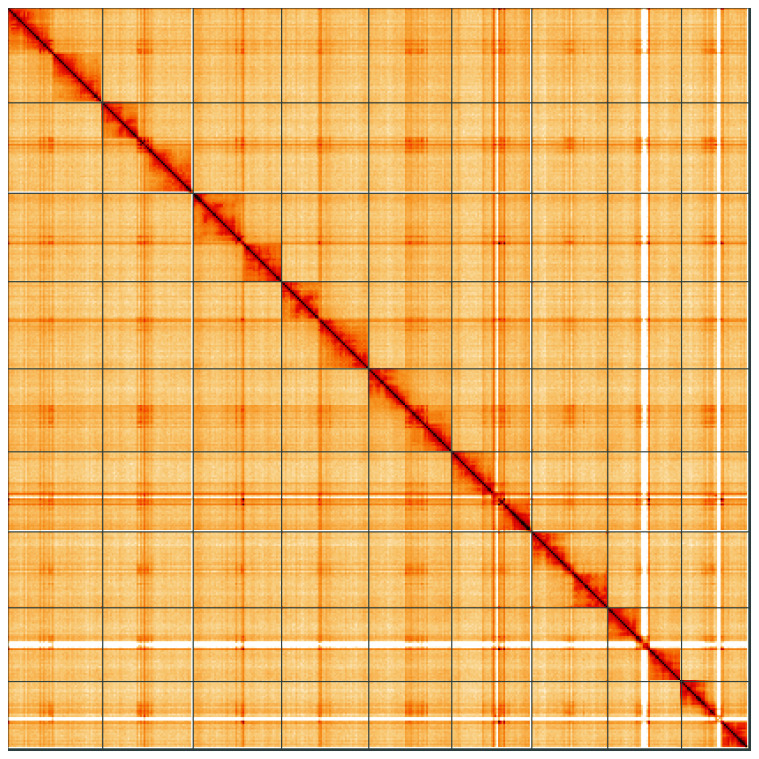
Genome assembly of *Lasioglossum pauxillum*, iyLasPaux1.1: Hi-C contact map of the iyLasPaux1.1 assembly, visualised using HiGlass. Chromosomes are shown in order of size from left to right and top to bottom. An interactive version of this figure may be viewed at https://genome-note-higlass.tol.sanger.ac.uk/l/?d=WN94LQFxSki1yrVGwhd-EA.

The final assembly has a total length of 432.0 Mb in 35 sequence scaffolds with a scaffold N50 of 48.2 Mb ( Table 1). The snailplot in Figure 2 provides a summary of the assembly statistics, while the distribution of assembly scaffolds on GC proportion and coverage is shown in Figure 3. The cumulative assembly plot in Figure 4 shows curves for subsets of scaffolds assigned to different phyla. Most (99.57%) of the assembly sequence was assigned to 9 chromosomal-level scaffolds, representing 9 autosomes. Chromosome-scale scaffolds confirmed by the Hi-C data are named in order of size ( Figure 5; Table 2). While not fully phased, the assembly deposited is of one haplotype. Contigs corresponding to the second haplotype have also been deposited. The mitochondrial genome was also assembled and can be found as a contig within the multifasta file of the genome submission.

**Table 1.** Genome data for *Lasioglossum pauxillum*, iyLasPaux1.1.

Project accession data		
Assembly identifier	iyLasPaux1.1	
Species	*Lasioglossum pauxillum*	
Specimen	iyLasPaux1	
NCBI taxonomy ID	88516	
BioProject	PRJEB51035	
BioSample ID	SAMEA7520494	
Isolate information	iyLasPaux1, female	
Assembly metrics *		*Benchmark*
Consensus quality (QV)	63.5	*≥ 50*
*k*-mer completeness	100.0%	*≥ 95%*
BUSCO **	C:95.7%[S:95.3%,D:0.5%],F:1.4%,M:2.9%,n:5,991	*C ≥ 95%*
Percentage of assembly mapped to chromosomes	99.57%	*≥ 95%*
Sex chromosomes	None	*localised homologous pairs*
Organelles	Mitochondrial genome: 27.71 kb	*complete single alleles*
Raw data accessions		
PacificBiosciences SEQUEL II	ERR9081702	
Hi-C Illumina	ERR8702822	
Genome assembly		
Assembly accession	GCA_933228785.1	
*Accession of alternate haplotype*	GCA_933228795.1	
Span (Mb)	432.0	
Number of contigs	159	
Contig N50 length (Mb)	10.7	
Number of scaffolds	35	
Scaffold N50 length (Mb)	48.2	
Longest scaffold (Mb)	55.06	
Genome annotation		
Number of protein-coding genes	12,353	
Number of non-coding genes	4,490	
Number of gene transcripts	30,008	

* Assembly metric benchmarks are adapted from column VGP-2020 of “Table 1: Proposed standards and metrics for defining genome assembly quality” from ( Rhie *et al.*, 2021). ** BUSCO scores based on the hymenoptera_odb10 BUSCO set using version 5.3.2. C = complete [S = single copy, D = duplicated], F = fragmented, M = missing, n = number of orthologues in comparison. A full set of BUSCO scores is available at https://blobtoolkit.genomehubs.org/view/CAKOFU01/dataset/CAKOFU01/busco.

**Table 2.** Chromosomal pseudomolecules in the genome assembly of *Lasioglossum pauxillum*, iyLasPaux1.

INSDC accession	Chromosome	Length (Mb)	GC%
OW121699.1	1	55.06	39.5
OW121700.1	2	52.52	40.0
OW121701.1	3	51.31	39.5
OW121702.1	4	50.62	40.0
OW121703.1	5	48.16	39.5
OW121704.1	6	46.47	40.5
OW121705.1	7	44.09	39.5
OW121706.1	8	42.84	40.0
OW121707.1	9	39.08	43.5
OW121708.1	MT	0.03	14.0

The estimated Quality Value (QV) of the final assembly is 63.5 with *k*-mer completeness of 100.0%, and the assembly has a BUSCO v5.3.2 completeness of 95.7% (single = 95.3%, duplicated = 0.5%), using the hymenoptera_odb10 reference set ( *n* = 5,991).

Metadata for specimens, barcode results, spectra estimates, sequencing runs, contaminants and pre-curation assembly statistics are given at https://links.tol.sanger.ac.uk/species/88516.

## Genome annotation report

The *Lasioglossum pauxillum* genome assembly (GCA_933228785.1) was annotated using the Ensembl rapid annotation pipeline ( Table 1; https://rapid.ensembl.org/Lasioglossum_pauxillum_GCA_933228785.1/Info/Index). The resulting annotation includes 30,008 transcribed mRNAs from 12,353 protein-coding and 4,490 non-coding genes.

## Methods

### Sample acquisition and nucleic acid extraction

A female *Lasioglossum pauxillum* (specimen ID Ox000076, ToLID iyLasPaux1) was collected from Wytham Woods, Oxfordshire (biological vice-county Berkshire), UK (latitude 51.78, longitude –1.32) on 2019-07-18 by potting. The specimen was collected and identified by Liam Crowley (University of Oxford) and preserved on dry ice.

The workflow for high molecular weight (HMW) DNA extraction at the Wellcome Sanger Institute (WSI) includes a sequence of core procedures: sample preparation; sample homogenisation, DNA extraction, fragmentation, and clean-up. In sample preparation, the iyLasPaux1 sample was weighed and dissected on dry ice ( Jay *et al.*, 2023). Tissue from the whole organism was homogenised using a PowerMasher II tissue disruptor ( Denton *et al.*, 2023a). HMW DNA was extracted using the Automated MagAttract v1 protocol ( Sheerin *et al.*, 2023). The DNA was sheared into an average fragment size of 12–20 kb in a Megaruptor 3 system with speed setting 30 ( Todorovic *et al.*, 2023). Sheared DNA was purified by solid-phase reversible immobilisation ( Strickland *et al.*, 2023): in brief, the method employs a 1.8X ratio of AMPure PB beads to sample to eliminate shorter fragments and concentrate the DNA. The concentration of the sheared and purified DNA was assessed using a Nanodrop spectrophotometer and Qubit Fluorometer and Qubit dsDNA High Sensitivity Assay kit. Fragment size distribution was evaluated by running the sample on the FemtoPulse system.

Protocols developed by the Tree of Life laboratory are publicly available on protocols.io ( Denton *et al.*, 2023b).

### Sequencing

Pacific Biosciences HiFi circular consensus DNA sequencing libraries were constructed according to the manufacturers’ instructions. DNA sequencing was performed by the Scientific Operations core at the WSI on a Pacific Biosciences SEQUEL II instrument. Hi-C data were also generated from remaining tissue of iyLasPaux1 using the Arima2 kit and sequenced on the HiSeq X Ten instrument.

### Genome assembly, curation and evaluation

Assembly was carried out with Hifiasm ( Cheng *et al.*, 2021) and haplotypic duplication was identified and removed with purge_dups ( Guan *et al.*, 2020). The assembly was then scaffolded with Hi-C data ( Rao *et al.*, 2014) using YaHS ( Zhou *et al.*, 2023). The assembly was checked for contamination and corrected using the gEVAL system ( Chow *et al.*, 2016) as described previously ( Howe *et al.*, 2021). Manual curation was performed using gEVAL, HiGlass ( Kerpedjiev *et al.*, 2018) and Pretext ( Harry, 2022). The mitochondrial genome was assembled using MitoHiFi ( Uliano-Silva *et al.*, 2023), which runs MitoFinder ( Allio *et al.*, 2020) or MITOS ( Bernt *et al.*, 2013) and uses these annotations to select the final mitochondrial contig and to ensure the general quality of the sequence.

A Hi-C map for the final assembly was produced using bwa-mem2 ( Vasimuddin *et al.*, 2019) in the Cooler file format ( Abdennur & Mirny, 2020). To assess the assembly metrics, the *k*-mer completeness and QV consensus quality values were calculated in Merqury ( Rhie *et al.*, 2020). This work was done using Nextflow ( Di Tommaso *et al.*, 2017) DSL2 pipelines “sanger-tol/readmapping” ( Surana *et al.*, 2023a) and “sanger-tol/genomenote” ( Surana *et al.*, 2023b). The genome was analysed within the BlobToolKit environment ( Challis *et al.*, 2020) and BUSCO scores ( Manni *et al.*, 2021; Simão *et al.*, 2015) were calculated.

Table 3 contains a list of relevant software tool versions and sources.

**Table 3.** Software tools: versions and sources.

Software tool	Version	Source
BlobToolKit	4.1.7	https://github.com/blobtoolkit/blobtoolkit
BUSCO	5.3.2	https://gitlab.com/ezlab/busco
Hifiasm	0.16.1-r375	https://github.com/chhylp123/hifiasm
HiGlass	1.11.6	https://github.com/higlass/higlass
Merqury	MerquryFK	https://github.com/thegenemyers/MERQURY.FK
MitoHiFi	2	https://github.com/marcelauliano/MitoHiFi
PretextView	0.2	https://github.com/wtsi-hpag/PretextView
purge_dups	1.2.3	https://github.com/dfguan/purge_dups
sanger-tol/genomenote	v1.0	https://github.com/sanger-tol/genomenote
sanger-tol/readmapping	1.1.0	https://github.com/sanger-tol/readmapping/tree/1.1.0
YaHS	yahs-1.1.91eebc2	https://github.com/c-zhou/yahs

### Genome annotation

The Ensembl gene annotation system ( Aken *et al.*, 2016) was used to generate annotation for the *Lasioglossum pauxillum* assembly (GCA_933228785.1). Annotation was created primarily through alignment of transcriptomic data to the genome, with gap filling via protein-to-genome alignments of a select set of proteins from UniProt ( UniProt Consortium, 2019).

### Wellcome Sanger Institute – Legal and Governance

The materials that have contributed to this genome note have been supplied by a Darwin Tree of Life Partner. The submission of materials by a Darwin Tree of Life Partner is subject to the **‘Darwin Tree of Life Project Sampling Code of Practice’**, which can be found in full on the Darwin Tree of Life website here. By agreeing with and signing up to the Sampling Code of Practice, the Darwin Tree of Life Partner agrees they will meet the legal and ethical requirements and standards set out within this document in respect of all samples acquired for, and supplied to, the Darwin Tree of Life Project.

Further, the Wellcome Sanger Institute employs a process whereby due diligence is carried out proportionate to the nature of the materials themselves, and the circumstances under which they have been/are to be collected and provided for use. The purpose of this is to address and mitigate any potential legal and/or ethical implications of receipt and use of the materials as part of the research project, and to ensure that in doing so we align with best practice wherever possible. The overarching areas of consideration are:

•   Ethical review of provenance and sourcing of the material

•   Legality of collection, transfer and use (national and international)

Each transfer of samples is further undertaken according to a Research Collaboration Agreement or Material Transfer Agreement entered into by the Darwin Tree of Life Partner, Genome Research Limited (operating as the Wellcome Sanger Institute), and in some circumstances other Darwin Tree of Life collaborators.

## Data Availability

European Nucleotide Archive: *Lasioglossum pauxillum* (base-banded furrow bee). Accession number PRJEB51035; https://identifiers.org/ena.embl/PRJEB51035 ( Wellcome Sanger Institute, 2022). The genome sequence is released openly for reuse. The *Lasioglossum pauxillum* genome sequencing initiative is part of the Darwin Tree of Life (DToL) project. All raw sequence data and the assembly have been deposited in INSDC databases. Raw data and assembly accession identifiers are reported in Table 1.

## Author information

Members of the University of Oxford and Wytham Woods Genome Acquisition Lab are listed here: https://doi.org/10.5281/zenodo.7125292.

Members of the Darwin Tree of Life Barcoding collective are listed here: https://doi.org/10.5281/zenodo.4893703.

Members of the Wellcome Sanger Institute Tree of Life Management, Samples and Laboratory team are listed here: https://doi.org/10.5281/zenodo.10066175.

Members of Wellcome Sanger Institute Scientific Operations: Sequencing Operations are listed here: https://doi.org/10.5281/zenodo.10043364.

Members of the Wellcome Sanger Institute Tree of Life Core Informatics team are listed here: https://doi.org/10.5281/zenodo.10066637.

Members of the Tree of Life Core Informatics collective are listed here: https://doi.org/10.5281/zenodo.5013541.

Members of the Darwin Tree of Life Consortium are listed here: https://doi.org/10.5281/zenodo.4783558.
