# Supplementary material for: The genome sequence of the Lobe-spurred Furrow Bee, Lasioglossum pauxillum (Schenck, 1853)
Source: Wellcome Open Res. 2024 Feb 19;9:86. [Version 1] doi: 10.12688/wellcomeopenres.20950.1 (PMC11339594; doi:10.12688/wellcomeopenres.20950.1)
Supplement: Peer review history

# Reviewer report: Reviewer response for version 1

**DOI:** 10.21956/wellcomeopenres.23177.r93784

**Published:** 2024-08-26

**Authors:** Daniel Garcia-Souto

**Affiliations:** 1 University of Santiago de Compostela, Galicia, Spain

The manuscript presents a high-quality genome assembly of a female of *Lasioglossum pauxillum*, utilizing cutting-edge sequencing techniques and thorough validation methods. The assembly achieved 59-fold coverage with Pacific Biosciences HiFi long reads and was scaffolded with Hi-C data, resulting in 35 sequence scaffolds with an N50 of 48.2 Mb. This represents a significant improvement over the previous genome assembly for this species (GCA_028455745), which was not referenced in the manuscript (and likely should have). Although initially, the scaffold count exceeded the species' known diploid number (2n=18), a subsequent manual curation reduced the scaffold numbers by 52.05% and doubled the scaffold N50, achieving a complete chromosome-level assembly. Thus, the final assembly is nearly complete, with 99.57% of the sequence assigned to nine chromosomal scaffolds and a BUSCO completeness score of 95.7%. This work represents a valuable genomic resource for the entomology community.

Are sufficient details of methods and materials provided to allow replication by others?

Yes

Is the rationale for creating the dataset(s) clearly described?

Yes

Are the datasets clearly presented in a useable and accessible format?

Yes

Are the protocols appropriate and is the work technically sound?

Yes

Reviewer Expertise:

Molecular cytogenetics, Genome assembly, Gene Annotation, Repetitive DNA

I confirm that I have read this submission and believe that I have an appropriate level of expertise to confirm that it is of an acceptable scientific standard.

# Reviewer report: Reviewer response for version 1

**DOI:** 10.21956/wellcomeopenres.23177.r93786

**Published:** 2024-08-21

**Authors:** Ruiqi Li

**Affiliations:** 1 University of Colorado Boulder, Boulder, Colorado, USA

Crowley et al. have presented a high-quality genome of the Lobe-spurred Furrow Bee, *Lasioglossum pauxillum*. I have a few minor comments.

**Figure 1:** While a fluidX tube is used as a scale, this may not be familiar to all readers. I suggest adding a scale reference based on the tube’s dimensions.

**Introduction**: The authors have provided a strong overview of the species’ ecology. However, the transition to the potential uses of the genome feels abrupt. A smoother transition from the species’ biology to the relevance of its genome would improve readability. For instance, introducing the topic of sociality before discussing the genome's role in studying the evolution of sociality would be beneficial.

**Genome Sequence Report:** The genome quality appears to be very high—well done!

**Methods:** Could the authors specify how RNA was extracted and from which tissues?

 The chromosome count in *Lasioglossum pauxillum* seems lower compared to other *Lasioglossum* species, such as *Lasioglossum* lativentre, which has 14 chromosomes. I recommend the authors double-check their manual curation of the chromosome count.

Are sufficient details of methods and materials provided to allow replication by others?

Yes

Is the rationale for creating the dataset(s) clearly described?

Yes

Are the datasets clearly presented in a useable and accessible format?

Yes

Are the protocols appropriate and is the work technically sound?

Yes

Reviewer Expertise:

Genomics

I confirm that I have read this submission and believe that I have an appropriate level of expertise to confirm that it is of an acceptable scientific standard.

## References

[1] : The genome sequence of the giant clam, Tridacna gigas (Linnaeus, 1758). *Wellcome Open Res* .2024;9: 10.12688/wellcomeopenres.21136.1 145 10.12688/wellcomeopenres.21136.1 38800516 PMC11116938

[2] : The genome sequence of the furry-claspered furrow bee, Lasioglossum lativentre (Schenck, 1853). *Wellcome Open Res* .2022;7: 10.12688/wellcomeopenres.17706.1 57 10.12688/wellcomeopenres.17706.1 36865377 PMC9971656

# Reviewer report: Reviewer response for version 1

**DOI:** 10.21956/wellcomeopenres.23177.r87631

**Published:** 2024-08-01

**Authors:** Jonathan Berenguer Uhuad Koch

**Affiliations:** 1 USDA-ARS Pollinating Insect Research Unit, Logan, Utah, USA

The author presents a high quality genome sequence of Lasioglossum pauxillum (Schenck, 1853). The author provides all the sufficient information of the materials, sequencing details, and bioinformatics tools used to achieve a highly contiguous genome of L. pauxillum. I am quite intrigued by the size of the genome of the L. pauxillum. While not required, I am curious to learn how the size of this bee's genome compares to other bee genome sequences.

Are sufficient details of methods and materials provided to allow replication by others?

Yes

Is the rationale for creating the dataset(s) clearly described?

Yes

Are the datasets clearly presented in a useable and accessible format?

Yes

Are the protocols appropriate and is the work technically sound?

Yes

Reviewer Expertise:

population genetics, genomics, entomology, melittology

I confirm that I have read this submission and believe that I have an appropriate level of expertise to confirm that it is of an acceptable scientific standard.
